# Transcriptome analysis of pigeon milk production – role of cornification and triglyceride synthesis genes

**DOI:** 10.1186/1471-2164-14-169

**Published:** 2013-03-13

**Authors:** Meagan J Gillespie, Tamsyn M Crowley, Volker R Haring, Susanne L Wilson, Jennifer A Harper, Jean S Payne, Diane Green, Paul Monaghan, John A Donald, Kevin R Nicholas, Robert J Moore

**Affiliations:** 1 Australian Animal Health Laboratory, CSIRO Animal, Food and Health Sciences, 5 Portarlington Road, Geelong, Victoria, Australia; 2 School of Life and Environmental Sciences, Deakin University, Pigdons Road, Geelong, Victoria 3216, Australia; 3 Centre for Chemistry and Biotechnology, Deakin University, Geelong, Victoria 3216, Australia

## Abstract

**Background:**

The pigeon crop is specially adapted to produce milk that is fed to newly hatched young. The process of pigeon milk production begins when the germinal cell layer of the crop rapidly proliferates in response to prolactin, which results in a mass of epithelial cells that are sloughed from the crop and regurgitated to the young. We proposed that the evolution of pigeon milk built upon the ability of avian keratinocytes to accumulate intracellular neutral lipids during the cornification of the epidermis. However, this cornification process in the pigeon crop has not been characterised.

**Results:**

We identified the epidermal differentiation complex in the draft pigeon genome scaffold and found that, like the chicken, it contained beta-keratin genes. These beta-keratin genes can be classified, based on sequence similarity, into several clusters including feather, scale and claw keratins. The cornified cells of the pigeon crop express several cornification-associated genes including *cornulin*, *S100-A9* and *A16-like*, *transglutaminase 6-like* and the pigeon ‘lactating’ crop-specific annexin *cp35*. Beta-keratins play an important role in ‘lactating’ crop, with several claw and scale keratins up-regulated. Additionally, *transglutaminase 5* and differential splice variants of *transglutaminase 4* are up-regulated along with *S100-A10*.

**Conclusions:**

This study of global gene expression in the crop has expanded our knowledge of pigeon milk production, in particular, the mechanism of cornification and lipid production. It is a highly specialised process that utilises the normal keratinocyte cellular processes to produce a targeted nutrient solution for the young at a very high turnover.

## Background

Pigeon lactation was first noted in the literature in 1786 when John Hunter described pigeon milk as being like “..granulated white curd” [1]. This curd-like substance is produced in the crop of male and female pigeons and regurgitated to the young. Like the mammary gland, the pigeon crop undergoes significant changes to the tissue structure during lactation. Several histological studies have characterised these changes and determined that pigeon milk consists of desquamated, sloughed crop epithelial cells [2,3]. The process of pigeon milk production begins when the germinal cell layer of the crop rapidly proliferates in response to prolactin [4,5], and this results in a convoluted, highly folded epithelial structure that then coalesces as it out-grows the vasculature, to form the nutritive cell layer that is sloughed off to produce the milk. This nutritive cell layer contains lipid-filled vacuoles [2,3,5,6]. The lipid content of pigeon milk consists mainly of triglycerides, along with phospholipids, cholesterol, free fatty acids, cholesterol esters and diglycerides [7]. The triglyceride content decreases across the lactation period, from 81.2% of total lipid at day one, to 62.7% at day 19, whereas the other lipids increase, which suggests the cellular lipid content decreases towards the end of the lactation period, but the cell membrane-associated lipids remain constant [7].

Several studies have investigated the differences in gene expression between ‘lactating’ pigeon crop tissue and non-‘lactating’ crop tissue [6,8,9]. Nearly three decades ago, Horseman and Pukac were the first to identify that mRNA species differ in response to prolactin injection in the crop [8]. Specifically, they identified and characterised gene expression and protein translation of the prolactin-responsive mRNA *anxI*_*cp35*_ and the non-prolactin-responsive isoform, *anxI*_*cp37*_[9,10]. In addition, a recent global gene expression study in our laboratory [6] showed that genes encoding products involved in triglyceride synthesis and tissue signalling were up-regulated in the ‘lactating’ crop. We proposed that the evolution of the processes that result in the production of pigeon milk has built upon the more general ability of avian keratinocytes to accumulate intracellular neutral lipids during the cornification of the epidermis [11] in order to produce a nutritive substance for their young [6].

The mechanism of avian epidermal cornification and lipid accumulation is not well-characterised. However, studies have shown that antibodies against mammalian cornification proteins, which are relatively well-characterised, can cross-react with avian and reptilian species [12,13], which suggests similarities in cornification proteins amongst vertebrate species. Cultured chicken keratinocytes have been shown to express beta-keratins (feather, scale and claw keratins), alpha-keratins (type I and II cytokeratins) and the cornified envelope precursor genes envoplakin and periplakin, as well as accumulating neutral lipids [11]. Mammalian keratinocytes differ from avian keratinocytes in that they are unable to accumulate intracellular neutral lipids [11], and can express alpha-keratins (cytokeratins) but not beta-keratins, which expanded from early archosaurians [14]. There are many cornification-associated proteins characterised from mammalian epidermal tissues. The proteins that form the cornified envelope include keratins, S100 proteins, small proline-rich proteins (SPRRs), late cornified envelope (LCE) proteins, annexins, involucrin, loricrin, filaggrin, desmoplakin, envoplakin, periplakin, trichohyalin, cystatin A, elafin and repetin [15]. Trans-glutaminase enzymes, some of which require cleavage by proteases and an increase in intracellular calcium concentration to become active, cross-link the cornified envelope proteins to form a ceramide lipid-coated protective barrier to the epidermis [16]. Many of the cornified envelope genes are present in the “epidermal differentiation complex” (EDC) which was first identified on chromosome 1q21 in humans [17]. Interestingly, the EDC region has been identified in an avian species (chicken), and is linked to the genes for beta-keratins, but lacks the LCE proteins [18].

Here we present an analysis of the pigeon crop transcriptome to show that pigeon milk production involves a specialised cornification process and *de novo* synthesis of lipids that accumulate intracellularly.

## Results

### Differentiation of the ‘lactating’ crop

**Figure 1:**
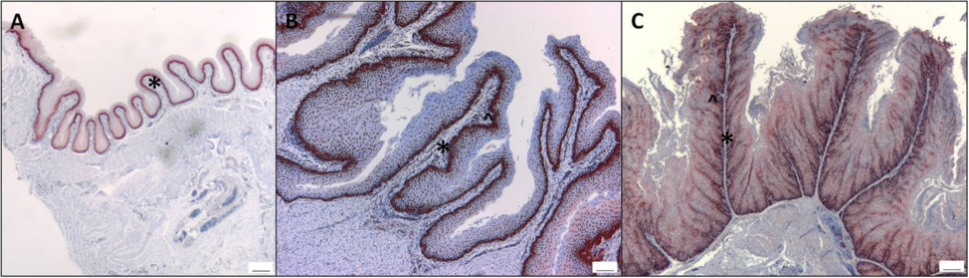
**The pigeon crop differentiates during nesting.** The non-‘lactating’ cropsac (**A**) differentiates during nesting (**B**&**C**), such that the lamina propria (*) becomes progressively more extended and narrow, and the number and depth of rete pegs (^) increases as the cropsac further differentiates. Proliferating cells are stained red with an antibody to proliferating cell nuclear antigen, and non-proliferating cells are counterstained blue with hematoxylin. Scale bar = 100 μm.

**Figure 2:**
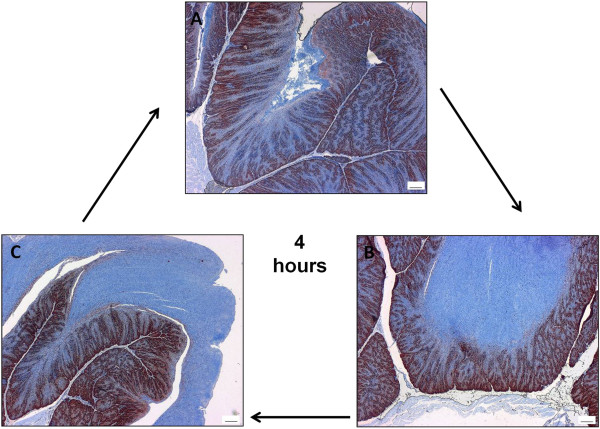
**Pigeon milk production is a four hour cycle.** Pigeon milk is produced by proliferation of the crop germinal epithelium (proliferating cells of the germinal epithelium are stained red with an antibody to proliferating cell nuclear antigen) which results in the accumulation of the differentiated lipid and protein containing cells (counterstained blue with hematoxylin) every 4 hours during lactation. (**A**) The crop ready to regenerate pigeon milk after it has been regurgitated to the young. (**B**) Pigeon milk (blue) has been produced and is nearly ready to separate from the germinal epithelium. (**C**) The pigeon ‘milk has started to separate from the germinal epithelium, ready for regurgitation to the young. Scale bar = 100 μm.

Immunohistological analysis of the proliferating cells of the pigeon crop in its resting state and during nesting demonstrated the morphological changes that occur in preparation for pigeon milk production (Figure 1). As the crop changed in preparation for lactation, the number and depth of rete pegs increased and the lamina propria became progressively more extended and narrow, which increased the surface area of the crop. During lactation the crop was highly proliferative, which resulted in the accumulation and sloughing of large tracts of cornified epithelium (Figure 2). All lactating parents in this study (48 birds) fed their young every four hours over the lactation period. Histology revealed a cycle of production and turnover of cornified epithelium over the four-hour period (Figure 2). The squabs milk intake reduced gradually toward the end of the lactation period, which lasted approximately fourteen days.

Analysis of transcriptional changes over the lactation period compared to non-‘lactating’ crop revealed no differentially expressed probes at pre-hatch (time points −8 and −2), large differences at hatch (time point 0) and 2 days post-hatch (time point +2) (17.2 and 48.8% of all probes differentially expressed, respectively), and no difference above what could be expected by chance (5%) at 10 days post-hatch (time point +10) (2.7% of probes differentially expressed) (Additional file 1: Table S1). Any effect of sex was ruled out by comparing males to females at non-‘lactating’ and ‘lactating’ time points. There was no difference above what could be expected by chance (Additional file 1: Table S2).

### Cornification genes are differentially expressed in the ‘lactating’ pigeon crop

**Figure 3:**
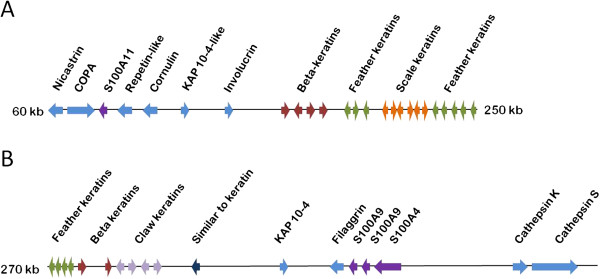
**The pigeon epidermal differentiation complex.** The pigeon EDC is located on (**A**) scaffolds 1246 and (**B**) 683 of the draft pigeon genome. It is bound by *nicastrin* and *cathepsin S*, and contains putative genes for the cornified envelope precursors repetin, cornulin, involucrin and filaggrin. In addition, the S100 genes *S100A11*, *S100A4* and two copies of *S100A9* are present. Two putative keratin-associated proteins (KAPs) are present, and clusters of beta keratins, feather keratins, scale keratins and claw keratins.

**Figure 4:**
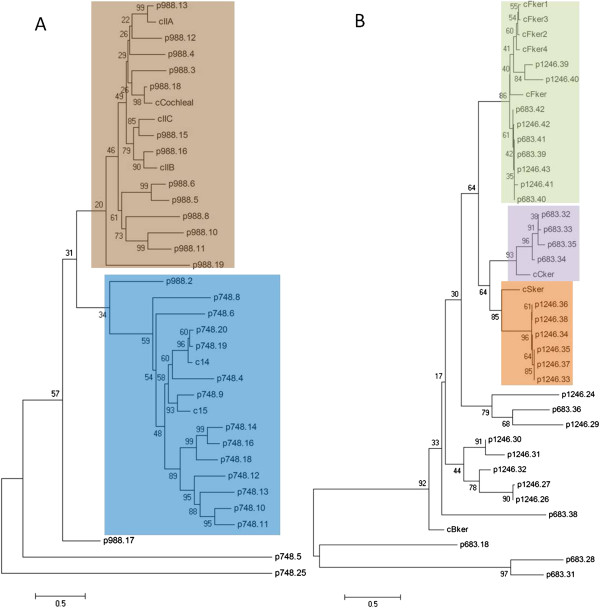
**Phylogenetic analysis of putative pigeon keratin proteins.** Neighbour-joining minimum evolution trees of putative pigeon (**A**) alpha and (**B**) beta keratins showing the relationship to chicken keratins are presented. Putative pigeon keratins are given the prefix p followed by the ORF number, and chicken keratins are given the prefix c. For a list of GenBank IDs corresponding to the chicken keratins refer to Additional file 2. (**A**) Putative pigeon alpha keratins form two distinct groups. The first consists of type II cytokeratins (brown) and the second consists of type I cytokeratins (blue). (**B**) Putative pigeon beta keratins form several distinct groups including groups of feather (green), claw (purple) and scale (orange) keratins. Additionally there are several smaller groups that contain no chicken homolog.

Analysis of cornification-associated genes in the draft pigeon genome identified an epidermal differentiation complex (EDC) on scaffolds 1246 and 683, respectively (Figure 3). Transcriptional analysis of these EDC genes and other cornification-associated genes in the pigeon crop at time points 0 and +2 revealed differential expression of 43 genes in 0, +2 or both ‘lactating’ pigeon crops compared with non-‘lactating’ crop (Table 1). Thirteen of these genes were up-regulated and 30 were down-regulated. Notably, the majority of cornification-associated genes up-regulated in the ‘lactating’ crop were keratins, constituting eight of the thirteen up-regulated genes. Five of these eight keratins were beta-keratins and three were alpha-keratins. Conversely, eight of the 30 down-regulated cornification-associated genes were alpha-keratins, and none were beta-keratins. Phylogenetic analysis of the beta-keratins (Figure 4), which were all part of the pigeon EDC (Figure 3) separates them into several groups. Feather, claw and scale keratins share a common ancestor related to chicken beta-keratin, from which feather keratins (none differentially expressed in ‘lactating’ crop) formed their own clade, and claw (ns and 10.5 to 12-fold up-regulated) and scale keratins (ns and 3.2-fold up-regulated) formed another monophyletic clade. Putative pigeon keratins formed three more clades not containing a chicken homolog, and ORF 683_38 formed a clade of its own. GenBank IDs of keratins with the highest amino acid identity to the pigeon keratins are found in Additional file 2.

**Table 1:** Differential expression of cornification-associated genes in the ‘lactating’ crop

**Gene**	**0/ctrl**	**+2/ctrl**
**Up-regulated genes**		
*cp35*	5.69	22.81
*similar to keratin (ORF 683_28)*	5.28	21.31
*claw keratin-like (ORF 683_34)*	ns	12.06
*claw keratin-like (ORF 683_32, 683_35)*	ns	10.53
*similar to keratin 19 (ORF 748_10)*	ns	3.92
*scale keratin-like (ORF 1246_34, 1246_36)*	ns	3.27
*similar to transglutaminase 5*	3.10	2.71
*cp37*	ns	2.70
*similar to keratin type II (ORF 988_5)*	ns	2.43
*scale keratin-like (ORF 683_38)*	ns	2.31
*Keratin, type II cytoskeletal 6A (ORF 988_4)*	ns	2.27
*Transglutaminase 4 exon 15*	1.21	1.35
*Transglutaminase 4 exon 14*	1.16	1.27
*Transglutaminase 4 exon 12, 13*	1.19	ns
*Transglutaminase 4 exon 6*	ns	1.30
*Transglutaminase 4 exon 3, 4*	1.71	2.38
*Protein S100-A10*	1.31	1.59
**Down-regulated genes**		
*s100-A14-like*	ns	−1.20
*Annexin A7*	−1.14	−1.25
*CystatinA*	ns	−1.31
*s100-A16-like*	ns	−1.33
*Desmoplakin*	−1.50	−1.54
*Annexin A5*	ns	−1.61
*Annexin A2*	−1.21	−1.65
*Sciellin*	ns	−1.83
*keratin 14 (ORF 748_19)*	ns	−1.88
*Periplakin*	ns	−1.88
*Annexin A11*	−1.39	−1.90
*Transglutaminase 6-like*	ns	−1.95
*Envoplakin*	−2.15	−2.00
*keratin, type II cytoskeletal cochleal-like (ORF 988_18)*	−1.45	−2.02
*keratin 80 (ORF 988_19)*	ns	−2.26
*Desmoplakin isoform 2*	−1.49	−2.36
*Epiplakin*	−1.55	−2.52
*Epiplakin-like*	−1.54	−2.59
*similar to keratin 5 (ORF 988_13)*	ns	−2.77
*Annexin A6*	−1.72	−2.85
*Annexin A8*	−1.73	−2.92
*protein S100-A9-like*	ns	−3.08
*keratin, type I cytoskeletal 23-like (ORF 748_8)*	−1.74	−3.38
*keratin, type I cytoskeletal 14-like (ORF 748_20)*	ns	−3.39
*Tissue transglutaminase*	−2.13	−4.22
*keratin, type I cytoskeletal 17-like (ORF 748_14)*	−2.42	−5.27
*keratin 12 (ORF 748_4)*	−3.16	−7.42
*similar to s100 calcium-binding protein A4*	−2.71	−8.41
*similar to s100 calcium-binding protein P*	−3.37	−10.19
*Annexin A13*	−4.27	−21.01
*Transglutaminase 4 exon 10, 11*	ns	−1.39
*Transglutaminase 4 exon 7*	−1.55	−1.56

Average fold-change of significantly (*p* < 0.05) differentially expressed cornification-associated genes in the ‘lactating’ pigeon crop as compared to the non-‘lactating’ crop. ns = not significant.

Phylogenetic analysis of the alpha-keratins separates them into type I and type II (Figure 4). Seven type I keratins and two type II keratins were down-regulated, and two type II keratins were up-regulated in ‘lactating’ crop (Table 1). Notably, all of the type I putative pigeon keratins were constrained to scaffold988, whereas the type I keratins included 15 putative genes on scaffold748 and two on scaffold988 (Figure 4). All of the chicken alpha-keratins had a closely related putative pigeon homolog.

Aside from keratins, there were several other differentially expressed cornification-associated genes in the pigeon crop. Up-regulated cornification-associated genes in the ‘lactating’ crop at 0 and +2 timepoints included *transglutaminase 5* (3.1 and 2.7-fold), *S100-A10* (1.3 and 1.6-fold) and the pigeon lactation-specific transcript, *cp35* (5.7 and 23-fold), and its isoform *cp37* (ns and 2.7-fold). Probes on exons 3, 4, 6, 12, 13, 14 and 15 of *transglutaminase 4* were up-regulated (1.2 to 1.7 and 1.3 to 2.4-fold), whereas probes on exons 7, 10 and 11 were down-regulated (−1.5 and −1.4 to −1.6-fold), which suggests it is likely to be alternatively spliced in the pigeon crop. The transglutaminase genes *transglutaminase 6-like* and *tissue transglutaminase* were also down-regulated by 2-fold and 2.1 to 4.2-fold, respectively. Several of the S100 genes were down-regulated in the ‘lactating’ crop by 1.2 to 10.2-fold, including *S100-A14-like*, *S100-A16-like*, *S100-A9-like*, *S100-A4* and *S100P* (Table 1). The annexin genes *annexin A2, A5, A6, A7, A8, A11* and *A13* were down-regulated by 1.1 to 21-fold (Table 1). The envelope precursor protein-encoding genes *desmoplakin*, *periplakin*, *envoplakin*, *epiplakin* and *sciellin* and *cystatin A* were all down-regulated in the ‘lactating’ crop by 1.3 to 2.6-fold (Table 1).

The proteases *calpain-15* (ns and 6.6-fold) and *calpain 9 isoform 1* (ns and 1.8-fold) were up-regulated in ‘lactating’ crop, whilst *calpain-5* was down-regulated by 2.4 to 4.4-fold. *Cathepsins H*, *C*, *B-like*, *S*, *Z*, *L* and *D* were all down-regulated by between 1.2 and 1.8-fold.

The epithelial cell-derived antimicrobial peptide encoding gene *beta defensin 5* was up-regulated by 2.3 to 4.8-fold in ‘lactating’ crop at timepoints 0 and +2, respectively (Additional file 3).

### Differential expression of cornification-associated genes in the cornified epithelial cells of the pigeon crop

Analysis of differentially expressed cornification-associated genes revealed that six genes were up-regulated and none were down-regulated in the cornified cell layer compared to the underlying cell layers (Table 2). The most highly up-regulated gene was *cornulin* (1004-fold up-regulated), followed by *cp35* (20-fold up-regulated). ly, *transglutaminase 6-like* (15-fold), *annexin A8* (4-fold), *S100-A9-like* (3.5-fold), and *S100-A16-like* (2-fold) were up-regulated in cornified cells.

**Table 2:** Differential expression of cornification-associated genes in the cornified cell layer of the ‘lactating’ crop

**Gene**	**A/B**	***p *value**
*Cornulin*	1004.57	0.038
*Annexin A8*	3.98	0.017
*Cp35*	20.20	0.008
*s100-A16-like*	2.07	0.030
*protein S100-A9-like*	3.54	0.046
*Transglutaminase 6-like*	14.98	0.006

Fold-change of significantly (*p* < 0.05) differentially expressed cornification-associated genes in the cornified cell layer (A) of the ‘lactating’ pigeon crop as compared to basal cells (B) from the same crop. n = 2.

### Triglyceride synthesis is up-regulated in the ‘lactating’ pigeon crop

**Figure 5:**
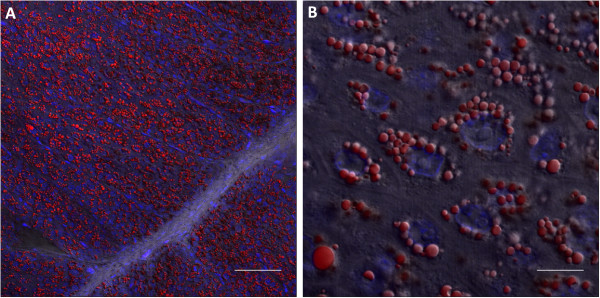
**Lipids form multiple intracellular droplets in the ‘lactating’ crop.** A fluorescent Oil Red O stain visualised by confocal microscopy reveals the location of neutral lipid in the ‘lactating’ pigeon crop. (**A**) Neutral lipid (red) is present throughout the differentiated epithelium of the ‘lactating’ pigeon crop. Scale bar = 60 μm. (**B**) Lipid droplets are perinuclear (nucleus stained blue, DAPI). Scale bar = 10 μm.

Examination of lipid droplets in ‘lactating’ pigeon crop showed that lipid was present throughout the differentiated epithelium, and was perinuclear (Figure 5). To investigate whether lipids could potentially be synthesised *de novo*, the expression of genes linked to milk lipid synthesis in the mouse mammary gland [19] were examined in the ‘lactating’ pigeon crop. Thirty-four mouse mammary gland-linked lipid synthesis genes were differentially expressed in the pigeon crop, including 7 variants of genes investigated in the mouse study. Expression patterns of milk lipid synthesis genes were similar in pigeon crop and mouse mammary gland, although pigeon crop expressed different variants of many genes of the triglyceride synthesis and fatty acid synthesis pathways in comparison to the mouse mammary gland. In particular, the triglyceride synthesis genes *Agpat1* and *Dgat1* were up-regulated in the lactating mouse mammary gland compared to pregnant mouse mammary gland [19], whereas *Agpat3*, *Agpat9* and *Dgat2* were up-regulated in the ‘lactating’ pigeon crop compared to non-‘lactating’ crop. The fatty acid synthesis gene *Elovl1* was up-regulated in lactating mouse [19], whereas *Elovl6* was up-regulated in ‘lactating’ pigeon crop. The lactating mouse mammary gland showed up-regulation of 5 different *Fabp* gene variants, whereas the ‘lactating’ pigeon crop up-regulated only *Fabp5*. Both lactating mouse and pigeon crop showed up-regulation of the same fatty acid transporter gene, *Slc27a4*, the fatty acid translocase, *Cd36*, and down-regulation of fatty acid transporter *Slc27a1*.

## Discussion

This is the first genome-wide pigeon crop transcriptome study to investigate the molecular mechanism of pigeon milk production. Here we show that differential expression of cornification-associated proteins and *de novo* lipid synthesis genes in the pigeon crop during lactation contribute to a highly specialised process that leads to the production of pigeon milk.

In preparation for lactation, the pigeon crop increases in surface area through an increase in rete pegs and extension of the lamina propria (Figure 1). This hyperplasia followed by desquamation results in large numbers of lipid-rich differentiated cells accumulating in the crop lumen, in the form of a curd-like substance, which provides nourishment for the young (Figure 2). Although the process of terminal differentiation, from the basal layer through to the desquamated layer takes days in mammals [20], it appears that the epidermal cells of the pigeon crop undergo a terminal differentiation program within the space of four hours. We have previously described this histologically [6]. The 1004-fold up-regulation of *cornulin* and 15-fold up-regulation of *transglutaminase 6* (Table 2), both late epidermal differentiation markers [21], in the cornified cell layer of the ‘lactating’ crop demonstrates the presence of terminally differentiated cells in the lactating pigeon crop epithelium.

Up-regulation of several beta keratins and three alpha keratins in the ‘lactating’ crop (Table 1) suggests an important function for keratin in the formation of pigeon milk. Beta-keratins are specific to archosaurians [22], and are found in the pigeon EDC (Figure 3), whereas alpha-keratins are ubiquitously expressed in eukaryotes. Phylogenetic analysis of the putative pigeon beta-keratins places the majority of up-regulated beta keratins in claw and scale beta keratin groups (Table 1, Figure 4). Beta-keratins have been suggested to have evolved from alpha-keratins to form a new class of matrix proteins that have a structural role in cornification [22]. Hence, it appears that in addition to alpha-keratins, beta-keratins have an important structural role in ‘lactating’ pigeon crop cells. Unlike alpha-keratins, beta-keratins form their own filament-matrix structures [23] which negates the need to express matrix proteins to form cornified beta-keratin epidermis. The down-regulation in ‘lactating’ crop of the typical mammalian cornified envelope precursors *desmoplakin*, *envoplakin*, *periplakin*, *sciellin* and *cystatin A* (Table 1) suggests that beta-keratins could play an alternative role to these matrix proteins in the ‘lactating’ crop.

Alpha-keratins are cross-linked to matrix proteins by transglutaminase enzymes, which are activated by proteolytic cleavage and increased intracellular calcium concentration [15]. S100 proteins play a role in the establishment of the calcium gradient in epithelial cell layers [24] and can also be substrates for transglutaminase themselves [25]. Both transglutaminase and S100 protein-encoding genes are up-regulated in ‘lactating’ crop (Table 1). Interestingly, *prostate transglutaminase* (*transglutaminase 4*) is differentially expressed in ‘lactating’ crop. Putative exons 7, 10 and 11 were down-regulated, while seven other putative exons were up-regulated, (Table 1) which suggests there could be multiple splice variants; this is the case for human *transglutaminase 4* in cancer tissues [26]. In addition, *transglutaminase 5*, which is expressed in mammalian cornifying epithelium [27] is up-regulated (Table 1). This is in contrast to mammalian cornifying tissues that express *transglutaminases 1* and *3* in addition to *transglutatminase 5*[28]. The up-regulation of the proteases *calpain-15* and *calpain 9 isoform 1* in the ‘lactating’ crop (Table 3) could suggest a role for these enzymes in the proteolytic activation of transglutaminases 4 and 5, as calpains are thought to activate transglutaminase 1 [15]. Additionally, cathepsin D has been suggested as an activator of transglutaminase, but this does not appear to be the case in the pigeon crop, as *cathepsin D* and six other cathepsin genes are down-regulated (Table 3).

**Table 3:** Differential expression of protease genes in the ‘lactating’ crop

**Gene**	**0/ctrl**	**+2/ctrl**
**Up-regulated genes**		
*Calpain-15*	ns	6.62
*Calpain 9 isoform 1*	ns	1.85
**Down-regulated genes**		
*Cathepsin H*	ns	−1.23
*Cathepsin C*	ns	−1.23
*Cathepsin B-like*	−1.19	−1.36
*Cathepsin S*	ns	−1.49
*Cathepsin Z*	ns	−1.50
*Cathepsin L*	ns	−1.53
*Cathepsin D*	−1.42	−1.76
*Calpain-5*	−2.39	−4.41

Average fold-change of significantly (*p* < 0.05) differentially expressed protease encoding genes in the ‘lactating’ pigeon crop as compared to the non-‘lactating’ crop. ns = not significant.

In addition to being a substrate for transglutaminase, S100 proteins can interact with annexins and form part of the cornified envelope [29]. S100-A10 is up-regulated in ‘lactating’ crop, as are the pigeon lactation-specific annexin gene *cp35* and its isoform *cp37* (Table 1), which could indicate roles for these genes in the formation of the cornified envelope. *Cp35* is expressed 20-fold higher in cornified cells of the ‘lactating’ crop, which suggests it has a function in the cornified cells, along with the S100 protein-encoding genes *S100-A16-like* and *S100-A9-like* (Table 2).

Accumulation of neutral lipids in keratinocytes is a unique trait of avian species [11]. The pigeon makes use of this ability in the crop to produce a lipid-rich milk [30] for the young. It was suggested by Garrison and Scow [31] that the lipids in pigeon milk were sequestered from another organ due to the increase in lipoprotein lipase activity in the prolactin-stimulated crop. We have also shown previously that there is up-regulation of genes involved in the oxidation of imported triglycerides [6], and in this study we found that lipoprotein lipase was up-regulated in ‘lactating’ crop (Table 4). However, the current study showed that lipid synthesis in the ‘lactating’ pigeon crop is a combination of importation and *de novo* synthesis of lipids, and results in the perinuclear accumulation of neutral lipid droplets (Figure 5).

**Table 4:** Differential expression of mammalian milk triglyceride synthesis genes in the ‘lactating’ crop

**Gene symbol**	**Gene name**	**0/ctrl**	**+2/ctrl**	**Mice L2/P7**
**Triglyceride synthesis**				
*Gyk*	*Glycerol kinase*	1.49	1.92	1.26
*Gpd1*	*Glycerol 3-PO4 dehydrogenase (cytosolic)*	−1.50	−2.08	2.25
*Acsl1*	*Long-chain Acyl-CoA synthetase 1*	1.80	2.94	1.51
*Acsl4*	*Long-chain-fatty-acid-CoA ligase 4*	ns	−2.42	−1.35
*Gpam; GPAT*	*Glycerol-3-phosphate acyltransferase*	ns	2.03	−1.22
*Agpat3*	1-acylglycerol-3-phosphate O-acyltransferase 3*	ns	1.52	Na
*Agpat9*	1-acylglycerol-3-phosphate O-acyltransferase 9*	ns	2.38	Na
*Dgat2*	Diacylglycerol O-acyltransferase 2*	1.61	3.66	Na
**Fatty acid plasma membrane transporters**				
*Slc27a1*	*Fatty acid transporter, member 1*	−2.21	−2.21	−1.01
*Slc27a4*	*Fatty acid transporter, member 4*	ns	2.01	1.99
*Cd36*	*Fatty acid translocase*	2.69	5.57	1.08
**Fatty acid binding proteins**				
*Fabp3*	*Fatty acid binding protein 3, muscle and heart*	−3.17	−4.88	−1.02
*Fabp5*	*Fatty acid binding protein 5*	ns	2.92	1.31
**Fatty acid degradation**				
*Lpl*	*Lipoprotein lipase*	1.66	2.08	1.24
*Cte1*	*Cytosolic acyl coenzyme A thioester hydrolase*	ns	1.59	−16.66
*Crat*	*Carnitine acetyltransferase IA*	ns	1.35	Na
*Crat*	*Carnitine acetyltransferase IB*	ns	−2.02	na
*Cpt2; CPTII*	*Carnitine acetyltransferase II*	ns	1.43	−2.56
*Ech1*	*Enoyl coenzyme A hydratase 1, peroxisomal*	ns	1.69	−2.08
*Hadhsc*	*L-3-hydroxyacyl-Coenzyme A dehydrogenase*	ns	1.49	−1.20
*LOC231086*	*Betaketothiolase*	ns	1.23	−1.56
**Fatty acid synthesis**				
*Acly*	*ATP citrate lyase*	1.46	1.88	2.66
*Mdh1*	*Malate dehydrogenase, cytosolic*	1.19	1.23	1.07
*Mod1*	*Malic enzyme, cytosolic*	1.41	2.17	1.54
*ACC*	*Acetyl CoA carboxylase*	1.68	2.26	Na
*Fasn*	*Fatty acid synthase*	1.60	2.46	1.65
*Scd1*	*Stearoyl-Coenzyme A desaturase 1*	ns	2.00	1.55^
*Scd5*	Stearoyl-Coenzyme A desaturase 5*	−1.45	−1.48	Na
*Fads1*	*Fatty acid desaturase 1*	ns	−2.22	2.40
*Elovl4*	Elongation of very long chain fatty acids 4*	ns	−7.28	Na
*Elovl5*	Elongation of very long chain fatty acids 5*	ns	−2.05	Na
*Elovl6*	Elongation of very long chain fatty acids 6*	1.32	1.63	Na
*Thrsp; Lpgp*	*Spot 14, thyroid hormone-inducible hepatic protein*	ns	−4.90	1.89

Average fold-change of significantly (*p* < 0.05) differentially expressed triglyceride synthesis genes in the ‘lactating’ pigeon crop at timepoint 0 and +2 as compared to the non-‘lactating’ crop. ns = not significant. Values for lactating mouse mammary gland versus pregnant [19] are given for comparison.

* = pigeon differentially expressed variant, not examined in mouse.

^ = average of 2 values.

Table 4 shows that genes involved in triglyceride synthesis in the mouse mammary gland during lactation [19] are also differentially expressed in the ‘lactating’ crop. The majority of genes involved in *de novo* lipid synthesis in the mouse are also expressed in the pigeon, but there are three gene variants that are expressed in the pigeon and not in the mouse. The pigeon expresses *Agpat3*, *Agpat9* and *Dgat* 2 (Table 4), whereas the mouse expresses *Agpat1* and *Dgat1*[19], which suggests that both the mechanism of lipid synthesis and crop cornification in the pigeon varies from that of mammals. The differences in the specific combinations of genes expressed may be reflected in the differences in triglycerides produced by each species. Amongst mammalian species there are differences in the fatty acid composition of milk triglycerides [32]. However, a comparison of the major fatty acid components of pigeon milk; oleic acid, linoleic acid and palmitic acid [30], reveals these are also the major fatty acid components of mammalian milk fat. There is a difference in the expression of *ELOVL* genes involved in fatty acid synthesis in the mouse mammary gland and in the pigeon crop (Table 4). In mouse and human, the *ELOVL1* gene is up-regulated during lactation [19,33], whereas the pigeon crop up-regulates *ELOVL6* during lactation (Table 4). It has been shown that *de novo* synthesis of fatty acids in the mammary gland can change in response to dietary availability [34]. Therefore, the difference in *ELOVL* gene expression between mammals and pigeons could be due to differences in the dietary availability of triglycerides/fatty acids in the pigeon diet. *ELOVL6,* up-regulated in ‘lactating’ crop, has been shown to have high elongation activity on C16:0 long chain fatty acids, and also some activity on C18:1 and C18:2 long chain fatty acids [35], which are the major fatty acid components of pigeon milk. This suggests that a large proportion of pigeon milk fatty acids could be synthesised *de novo* in the crop. One of the major differences between pigeon milk fatty acids and mammalian milk fatty acids is the lack of very long chain fatty acids, which are synthesised *de novo* by ELOVL1 [35].

Here we have shown that pigeon milk is the result of a specialised cornification process that produced large numbers of lipid-laden, cornified cells with a very rapid four hour cycle of hyperplasia followed by desquamation in the ‘lactating’ pigeon crop.

## Conclusions

This study has expanded our knowledge of pigeon milk production, in particular, the mechanism of cornification and lipid production in the crop. Pigeon lactation is a highly specialised process that utilises the normal keratinocyte cellular processes to produce a targeted nutrient solution for the young at a very high turnover rate.

## Methods

### Pigeon tissue sample collection

Thirty-two breeding pairs of King pigeons were purchased from Kooyong Squab Producers (Moama, New South Wales). They were housed in temperature-controlled cabinets (between 21–24°C) with a 12 hour light cycle (lights on 6 am), and supplied with nest bowls and nesting materials. Pigeons had *ad libitum* access to pigeon mix (pro-vit-min, Ivorsons, Geelong) and water. Control non-‘lactating’ pairs (ctrl, 13 birds) were culled prior to mating. Breeding pairs were culled at different lactation time points whereby squab hatch was designated as time zero. Time points pre-hatch have the prefix ‘-‘ and post-hatch have the prefix ‘+’. Specifically, breeding pairs were euthanised at 8 days pre-hatch (−8, n = 10 birds), 2 days pre-hatch (−2, n = 10 birds), at hatch (0, n = 14 birds), 2 days post-hatch (+2, n = 10 birds), and 10 days post-hatch (+10, n = 4 birds). Whole crop tissue samples were snap frozen in liquid nitrogen and separate samples of all crops were fixed in 10% neutral buffered formalin or snap frozen in optimal cutting temperature (OCT) compound for histology. Samples of pigeon crop from a time 0 pair were fixed in PaxGene (Qiagen) fixative according to the manufacturer’s instructions for laser dissection microscopy, to investigate gene expression differences between basal and proliferating cell types. Samples of other whole tissues (brain, pituitary, thymus, esophagus, trachea, proventriculus, gizzard, heart, kidney, duodenum, ileum, jejunum, pancreas, spleen, cecum, bone marrow, muscle and skin) were snap frozen in liquid nitrogen and used for the construction of a pooled tissues cDNA library. The blood and spleen of the ten day old squab were removed; the blood into vacutainers coated with EDTA dipotassium salt and the spleen into sterile media (DMEM with 10% FCS, 100 U/mL penicillin, 100 μg/mL streptomycin, 500 μg/mL fungizone).

All work using animals was conducted in accordance with the Australian Code of Practice for the Care and Use of Animals for Scientific Purposes (7th edition), and in accordance with institutional animal ethics guidelines (CSIRO AAHL Animal Ethics Committee).

### Pigeon splenocyte stimulation

The squab spleen was minced through a 70 μm filter using a syringe plunger into 15 mL phosphate buffered saline (PBS). The blood was diluted in PBS, and the cell suspensions were layered slowly over the same volume of Lymphoprep (Axis-Shield, Oslo, Norway). After centrifugation the cells were removed from the interface of the gradient and washed twice with 50 mL PBS + 10% FCS. The cells were seeded on a 24-well plate (Nunc) at 5 × 10^5^ cells/mL. To each well 10 μg/mL concanavalin A (Astral Scientific) was added and the cells were incubated at 37°C in 5% CO_2_. After 24 hours the cells were pelleted and re-suspended in 1 mL TRIreagant RT (Molecular Research Center) for RNA extraction and synthesis of the immune library.

### cDNA library synthesis

Double-stranded cDNA (dscDNA) libraries were syn-thesised from poly(A)^+^ RNA extracted from the female crop tissue samples and the pooled tissues and immune cells. Total RNA was extracted with TRIreagant RT (Molecular Research Center) according to the manufacturer’s instructions, with an additional high salt precipitation buffer step to remove glycoproteins. Poly(A)^+^ RNA was twice purified from total RNA using Dynal beads (Life Technologies), according to the manufacturer’s instructions. 200 ng poly(A)^+^ RNA was fragmented at 70°C for 30 s in a 20 μl reaction containing 18 μl fragmentation solution (100 mM Tris–HCl pH 7.0, 100 mM ZnCl_2_). The reaction was stopped on ice with 2 μl of 0.5 M EDTA pH 8.0 and 28 μl of 10 mM Tris–HCl pH 7.5 and cleaned up with an RNeasy column (Qiagen). The sequencing libraries were synthesised from the fragmented poly(A)^+^ RNA according to the Roche cDNA Rapid Library Preparation Method Manual, Rev. Jan 2010 beginning section 3.2. Briefly, fragmented RNA was reverse transcribed into cDNA and made double-stranded using a mixture of DNA polymerase I, ligase and RNase H and blunt ended with T4 DNA Polymerase (Roche cDNA synthesis system).

### High throughput sequencing, assembly, microarray design and annotation

Sequencing libraries were prepared from the dscDNA libraries using a Rapid Library Preparation Kit (Roche). Sequencing beads were generated with a SV-emPCR Kit (Roche) and sequenced on a 454 GS FLX using the titanium chemistry (Roche). Each sample was sequenced in a separate region. The raw reads from all regions were combined (430654) and assembled with Newbler v2.3 shotgun assembler (Roche). The resulting contigs (10463) and remaining Singletons (71997) were used to design unique microarray probes with OligoArray 2.1 [36]. The microarray probes were annotated via the source contigs or read sequences by a series of BLAST searches using an E-value of 10^-3^ as cut-off for all searches. The first search used BLASTX [37] with all sequences against a local copy of the non-redundant protein database (dated 11 April 2012). All non-matched sequences were then used in a BLASTN query [38] against the non-redundant nucleotide database (dated 13 April 2012). Finally, the remaining unmatched sequences were used as queries in a TBLASTX search [37] against the nucleotide database.

### Identification of putative pigeon cornification-associated full-length genes

Cornification-associated genes were identified by literature search, and Raw 454 reads or assembled contigs were used as local megaBLAST [38] queries against the *Columba livia* draft genome [39] to identify in which scaffold each gene of interest was present. The scaffolds of interest were then submitted to a Hidden Markov Model gene prediction program (FGENESH, Softberry; http://linux1.softberry.com/berry.phtml) using parameters for chicken (aves) to identify predicted full-length gene sequences. Where scaffolds were too large to be processed by FGENESH, the region of the scaffold with the BLAST match was submitted. Microarray probes were mapped to the predicted gene sequence by local BLAST. Non-redundant, non-overlapping microarray probes matching predicted gene coding sequences were identified by megaBLAST against the predicted gene sequences and the *Columba livia* draft genome sequence.

### Phylogenetic analysis of pigeon alpha and beta keratins

Phylogenetic trees were constructed separately for alpha- and beta-keratins. The evolutionary relatedness was inferred using the Minimum Evolution method [40]. The percentage of replicate trees in which the associated taxa clustered together in the bootstrap test (1000 replicates) was calculated [41]. The tree was drawn to scale, with branch lengths in the same units as those of the evolutionary distances used to infer the phylogenetic tree. The evolutionary distances were computed using the JTT matrix-based method [42] and are in the units of the number of amino acid substitutions per site. The ME tree was searched using the Close-Neighbor-Interchange (CNI) algorithm [43] at a search level of 1. The Neighbor-joining algorithm [44] was used to generate the initial tree. All positions containing alignment gaps and missing data were eliminated in pairwise sequence comparisons (Pairwise deletion option). Phylogenetic analyses were conducted in MEGA4 [45] after alignment in ClustalX [46].

### Laser dissection microscopy and RNA amplification

PaxGene fixed time 0 pigeon crop of a female and male breeding pair were dehydrated through fresh ethanol and xylene using an automated processor (Leica), and embedded in paraffin according to the PaxGene manufacturer’s instructions. Sections of 4 μm were cut by microtome and floated on to laser dissection slides (Leica #11505158 membrane slides PEN-membrane 2 um). The cornified crop epithelial cells and the basal cells of 5 serial sections of each crop were laser dissected using a Leica LMD6000 machine and collected by gravity into 500 μl PCR tubes. The dissected cells were dissolved in QIAzol by pipetting up and down, and RNA was extracted using the RNeasy Lipid Tissue kit according to the manufacturer’s instructions, and eluted in 30 μl water. RNA was quantified using a Bioanalyzer RNA Pico chip, and an equal amount of RNA of each of the four samples was used for two rounds of RNA amplification using an Ambion MessageAmp II aRNA Amplification Kit, according to the manufacturer’s instructions.

### Microarray hybridisation, scanning and data pre-processing

RNA was extracted from whole frozen pigeon crop tissue according to the manufacturer’s instructions (Qiagen RNeasy Lipid Tissue kit). RNA quality and quantity was measured using a Bioanalyser RNA Pico chip and 5 μg of this RNA was used to synthesise first-strand cDNA with oligo_dt_ primer according to the manufacturer’s instructions (Invitrogen SuperScript SuperMix) which was then purified using a PCR purification kit (Qiagen). cDNA was synthesised and purified from whole crop RNA and from amplified laser dissected sample RNA. All cDNA samples were labelled with Cy3 using a Roche One-Color DNA Labelling Kit according to the manufacturer’s instructions. The labelled microarray probes were re-suspended with a sample tracking control and hybridisation buffer and loaded on 12-plex 135 k custom pigeon microarrays (ArrayExpress ID A-MEXP-2257). These were hybridised for 20 hours in a NimbleGen Hybridisation Station (Roche) at 42°C and then washed using the NimbleGen wash buffer kit (Roche) according to the manufacturer’s instructions. Each subarray was scanned at 2 μm on autogain with a NimbleGen MS200 microarray scanner (Roche). Sample tracking controls and control spots were used to autoalign a grid over each subarray using NimbleGen MS200 Software (Roche).

### Microarray normalisation and statistical analysis

Robust Multichip Average (RMA) analysis [47] was used to background correct and normalise spot signal intensity. To compare datasets hybridised to different slides, the data were subjected to the non-parametric CombatR algorithm to remove batch effects [48]. The datasets were exported into GeneSpring (Agilent) and differentially expressed genes were identified using an unpaired Welch t-test assuming unequal variances with a Benjamini and Hochberg post-hoc test, with a false discovery rate of *p* = 0.05. The comparison of cell layers from laser dissected RNA omitted the post-hoc test as there were only two samples per group. All microarray data has been deposited into ArrayExpress (accession numbers E-MTAB-1317 for whole crop and E-MTAB-1318 for laser dissected cell layers).

### Proliferating cell nuclear antigen immunohistochemistry

Formalin fixed pigeon crop was dehydrated through ethanol and xylene and embedded in paraffin. Sections of 4 μm were dewaxed in xylene and rehydrated through ethanol. Antigen retrieval was performed using the Dako PT Link (97°C for 30 min) while immersed in Target Retrieval Solution High pH (Dako). Following retrieval the sections were quenched with hydrogen peroxide. Sections were then incubated for 1 h with primary antibody (PCNA, 1:8000; Abcam ab29). This was followed by a Mouse linker (EnVision Flex+, Dako) for 15 minutes to enhance the staining. Horseradish peroxidase conjugated secondary antibody (Envision Flex+, Dako) was then applied for 20 minutes. Sections were stained with 3-amino- 9-ethylcarbazole (AEC) substrate chromogen (Dako) for 10 min, and counterstained with Lillie-Mayer’s haematoxylin.

### Oil Red O staining and confocal microscopy

70 μm sections of formalin-fixed ‘lactating’ crop tissue were sectioned by vibrating microtome (Leica VT1200S). Sections were stained with Oil Red O according to the method of Lillie and Ashburn [49] and nuclei were labelled with DAPI for 15 min. Following a water wash, sections were mounted with Vectashield (Abacus, Australia). Samples were imaged sequentially for each dye with a Leica SP5 confocal microscope (Leica Microsystems, Sydney).

## Competing interests

The authors declare that they have no competing interests.

## Authors’ contributions

Conceived the project: MG, TC, VH and RM. Contributed to the formulation of ideas: JD, KN, and PM. Carried out experimental work: MG, VH, JP, JH, DG and PM. Responsible for animal husbandry: SW. Analysed data: MG and VH. Wrote the manuscript: MG. All authors read and approved the final manuscript.

## Supplementary Material

Additional file 1: Table S1 All differentially expressed probes in lactating crop compared to control crop. **Table S2.** Number of probes differentially expressed in female lactating crop compared to male lactating crop at the same lactation timepoint. Click here for file

Additional file 2 **Putative pigeon ORFs identified from the draft pigeon genome scaffold.** This spreadsheet contains two sheets. The first sheet contains all of the putative pigeon ORFs identified, the corresponding BLAST annotation, ID of the best match and percent amino acid identity. The second sheet contains the IDs of the chicken keratins used to construct the phylogenetic tree of putative pigeon keratins (Figure 4). Click here for file

Additional file 3 **Differentially expressed transcripts in the ‘lactating’ crop.** This spreadsheet contains all of the annotation information for each microarray probe, and the expression foldchange of differentially expressed probes (*p* < 0.05) in pigeon crop at each time point, and in laser dissected differentiated crop cells compared to less differentiated crop cells. Click here for file

## References

[B1] Hunter J Owen R Observations on certain parts of the animal economy: inclusive of several papers from the Philosophical transactions, etc 1840 New Orleans: Haswell, Barrington, and Haswell

[B2] Litwer G Die Histologischen Veränderungen der Kropfwandung bei Tauben, zur Zeit der Bebrütung und Ausfütterung ihrer Jungen Z Zellforsch Mikrosk Anat 1926 695 722

[B3] Weber W Zur Histologie und Cytologie der Kropfmilchbildung der Taube Z Zellforsch Mikrosk Anat 1962 56 247 276 10.1007/BF00325118

[B4] Riddle O Bates RW Dykshorn S The preparation, identification and and assay of prolactin - a hormone of the anterior pituitary Am J Physiol 1933 105 191 216

[B5] Dumont JN Prolactin-induced cytologic changes in the mucosa of the pigeon crop during crop- "milk" formation Z Zellforsch Mikrosk Anat 1965 68 6 755 782 10.1007/BF00343930 5877243

[B6] Gillespie MJ Haring VR McColl KA Monaghan P Donald JA Nicholas KR Moore RJ Crowley TM Histological and global gene expression analysis of the 'lactating' pigeon crop BMC Genomics 2011 12 1 452 10.1186/1471-2164-12-452 21929790 PMC3191541

[B7] Desmeth M Vandeputte-Poma J Lipid composition of pigeon cropmilk—I. Total lipids and lipid classes Comp Biochem and Physiol Part B: Comparative Biochemistry 1980 66 1 129 133 10.1016/0305-0491(80)90094-2

[B8] Pukac LA Horseman ND Regulation of pigeon crop gene expression by prolactin Endocrinology 1984 114 5 1718 1724 10.1210/endo-114-5-1718 6714160

[B9] Pukac LA Horseman ND Regulation of cloned prolactin-inducible genes in pigeon crop Mol Endocrinol 1987 1 2 188 194 10.1210/mend-1-2-188 3331713

[B10] Horseman ND A prolactin-inducible gene product which is a member of the calpactin/lipocortin family Mol Endocrinol 1989 3 5 773 779 10.1210/mend-3-5-773 2526923

[B11] Vanhoutteghem A Londero T Ghinea N Djian P Serial cultivation of chicken keratinocytes, a composite cell type that accumulates lipids and synthesizes a novel beta-keratin Differentiation 2004 72 4 123 137 10.1111/j.1432-0436.2004.07204002.x 15157236

[B12] Alibardi L Toni M Localization and characterization of specific cornification proteins in avian epidermis Cells Tissues Organs 2004 178 4 204 215 10.1159/000083732 15812148

[B13] Alibardi L Toni M Immuno-cross reactivity of transglutaminase and cornification marker proteins in the epidermis of vertebrates suggests common processes of soft cornification across species J Exp Zool B Mol Dev Evol 2004 302 6 526 549 15468051 10.1002/jez.b.21016

[B14] Greenwold MJ Sawyer RH Genomic organization and molecular phylogenies of the beta (beta) keratin multigene family in the chicken (Gallus gallus) and zebra finch (Taeniopygia guttata): implications for feather evolution BMC Evol Biol 2010 10 148 10.1186/1471-2148-10-148 20482795 PMC2894828

[B15] Zeeuwen PL Epidermal differentiation: the role of proteases and their inhibitors Eur J Cell Biol 2004 83 11–12 761 773 15679120 10.1078/0171-9335-00388

[B16] Hoffner G Vanhoutteghem A Andre W Djian P Transglutaminase in epidermis and neurological disease or what makes a good cross-linking substrate Adv Enzymol Relat Areas Mol Biol 2011 78 97 160 22220473 10.1002/9781118105771.ch3

[B17] Mischke D Korge BP Marenholz I Volz A Ziegler A Genes encoding structural proteins of epidermal cornification and S100 calcium-binding proteins form a gene complex ("epidermal differentiation complex") on human chromosome 1q21 J Invest Dermatol 1996 106 5 989 992 10.1111/1523-1747.ep12338501 8618063

[B18] Vanhoutteghem A Djian P Green H Ancient origin of the gene encoding involucrin, a precursor of the cross-linked envelope of epidermis and related epithelia Proc Natl Acad Sci U S A 2008 105 40 15481 15486 10.1073/pnas.0807643105 18809918 PMC2563112

[B19] Rudolph MC McManaman JL Phang T Russell T Kominsky DJ Serkova NJ Stein T Anderson SM Neville MC Metabolic regulation in the lactating mammary gland: a lipid synthesizing machine Physiol Genomics 2007 28 3 323 336 17105756 10.1152/physiolgenomics.00020.2006

[B20] Morris R Argyris TS Epidermal cell cycle and transit times during hyperplastic growth induced by abrasion or treatment with 12-O-tetradecanoylphorbol-13-acetate Cancer Res 1983 43 10 4935 4942 6883343

[B21] Contzler R Favre B Huber M Hohl D Cornulin, a new member of the "fused gene" family, is expressed during epidermal differentiation J Invest Dermatol 2005 124 5 990 997 10.1111/j.0022-202X.2005.23694.x 15854041

[B22] Alibardi L Dalla Valle L Nardi A Toni M Evolution of hard proteins in the sauropsid integument in relation to the cornification of skin derivatives in amniotes J Anat 2009 214 4 560 586 10.1111/j.1469-7580.2009.01045.x 19422429 PMC2736123

[B23] Fraser RD Parry DA The structural basis of the filament-matrix texture in the avian/reptilian group of hard beta-keratins J Struct Biol 2011 173 2 391 405 10.1016/j.jsb.2010.09.020 20869443

[B24] Santamaria-Kisiel L Rintala-Dempsey AC Shaw GS Calcium-dependent and -independent interactions of the S100 protein family Biochem J 2006 396 2 201 214 10.1042/BJ20060195 16683912 PMC1462724

[B25] Ruse M Lambert A Robinson N Ryan D Shon KJ Eckert RL S100A7, S100A10, and S100A11 are transglutaminase substrates Biochemistry 2001 40 10 3167 3173 10.1021/bi0019747 11258932

[B26] Cho SY Choi K Jeon JH Kim CW Shin DM Lee JB Lee SE Kim CS Park JS Jeong EM Differential alternative splicing of human transglutaminase 4 in benign prostate hyperplasia and prostate cancer Exp Mol Med 2010 42 4 310 318 10.3858/emm.2010.42.4.031 20177144 PMC2859330

[B27] Candi E Oddi S Paradisi A Terrinoni A Ranalli M Teofoli P Citro G Scarpato S Puddu P Melino G Expression of transglutaminase 5 in normal and pathologic human epidermis J Invest Dermatol 2002 119 3 670 677 10.1046/j.1523-1747.2002.01853.x 12230511

[B28] Eckert RL Sturniolo MT Broome AM Ruse M Rorke EA Transglutaminases in epidermis Prog Exp Tumor Res 2005 38 115 124 15746532 10.1159/000084236

[B29] Robinson NA Lapic S Welter JF Eckert RL S100A11, S100A10, annexin I, desmosomal proteins, small proline-rich proteins, plasminogen activator inhibitor-2, and involucrin are components of the cornified envelope of cultured human epidermal keratinocytes J Biol Chem 1997 272 18 12035 12046 10.1074/jbc.272.18.12035 9115270

[B30] Shetty S Hegde SN Changes in lipids of pigeon “milk” in the first week of its secretion Lipids 1991 26 11 930 933 10.1007/BF02535979

[B31] Garrison MM Scow RO Effect of prolactin on lipoprotein lipase in crop sac and adipose tissue of pigeons Am J Physiol 1975 228 5 1542 1544 1130558 10.1152/ajplegacy.1975.228.5.1542

[B32] Dils RR Comparative aspects of milk fat synthesis J Dairy Sci 1986 69 3 904 910 10.3168/jds.S0022-0302(86)80480-5 3711414

[B33] Maningat PD Sen P Rijnkels M Sunehag AL Hadsell DL Bray M Haymond MW Gene expression in the human mammary epithelium during lactation: the milk fat globule transcriptome Physiol Genomics 2009 37 1 12 22 10.1152/physiolgenomics.90341.2008 19018045 PMC2661101

[B34] Barber MC Clegg RA Travers MT Vernon RG Lipid metabolism in the lactating mammary gland Biochim Biophys Acta 1997 1347 2–3 101 126 9295156 10.1016/s0005-2760(97)00079-9

[B35] Ohno Y Suto S Yamanaka M Mizutani Y Mitsutake S Igarashi Y Sassa T Kihara A ELOVL1 production of C24 acyl-CoAs is linked to C24 sphingolipid synthesis Proc Natl Acad Sci U S A 2010 107 43 18439 18444 10.1073/pnas.1005572107 20937905 PMC2973002

[B36] Rouillard JM Zuker M Gulari E OligoArray 2.0: design of oligonucleotide probes for DNA microarrays using a thermodynamic approach Nucleic Acids Res 2003 31 12 3057 3062 10.1093/nar/gkg426 12799432 PMC162330

[B37] Altschul SF Madden TL Schaffer AA Zhang J Zhang Z Miller W Lipman DJ Gapped BLAST and PSI-BLAST: a new generation of protein database search programs Nucleic Acids Res 1997 25 17 3389 3402 10.1093/nar/25.17.3389 9254694 PMC146917

[B38] Zhang Z Schwartz S Wagner L Miller W A greedy algorithm for aligning DNA sequences J Comput Biol 2000 7 1–2 203 214 10890397 10.1089/10665270050081478

[B39] Li C Zhang G Gilbert T Wang T Genomic data from the Domestic Pigeon (Columba livia) 2011 GigaScience http://dx.doi.org/10.5524/100007

[B40] Rzhetsky A Nei M A simple method for estimating and testing minimum-evolution trees Mol Biol Evol 1992 9 5 945

[B41] Felsenstein J Confidence limits on phylogenies: an approach using the bootstrap Evolution 1985 39 4 783 791 10.2307/2408678 28561359

[B42] Jones DT Taylor WR Thornton JM The rapid generation of mutation data matrices from protein sequences Comput Appl Biosci 1992 8 3 275 282 1633570 10.1093/bioinformatics/8.3.275

[B43] Nei M Kumar S Molecular Evolution and Phylogenetics 2000 New York: Oxford University Press

[B44] Saitou N Nei M The neighbor-joining method: a new method for reconstructing phylogenetic trees Mol Biol Evol 1987 4 4 406 425 3447015 10.1093/oxfordjournals.molbev.a040454

[B45] Tamura K Dudley J Nei M Kumar S MEGA4: Molecular Evolutionary Genetics Analysis (MEGA) software version 4.0 Mol Biol Evol 2007 24 8 1596 1599 10.1093/molbev/msm092 17488738

[B46] Larkin MA Blackshields G Brown NP Chenna R McGettigan PA McWilliam H Valentin F Wallace IM Wilm A Lopez R Clustal W and Clustal X version 2.0 Bioinformatics 2007 23 21 2947 2948 10.1093/bioinformatics/btm404 17846036

[B47] Irizarry RA Hobbs B Collin F Beazer-Barclay YD Antonellis KJ Scherf U Speed TP Exploration, normalization, and summaries of high density oligonucleotide array probe level data Biostatistics 2003 4 2 249 264 10.1093/biostatistics/4.2.249 12925520

[B48] Johnson WE Li C Rabinovic A Adjusting batch effects in microarray expression data using empirical Bayes methods Biostatistics 2007 8 1 118 127 10.1093/biostatistics/kxj037 16632515

[B49] Lillie RD Ashburn LL Supersaturated solutions of fat stains in dilute isopropanol for demonstration of acute fatty degeneration not shown by Herxheimer’s technique ArchsPath 1943 46 432

