# Correction: Transcriptome analysis of pigeon milk production – role of cornification and triglyceride synthesis genes

**DOI:** 10.1186/1471-2164-15-185

**Published:** 2014-04-01

**Authors:** Meagan J Gillespie, Tamsyn M Crowley, Volker R Haring, Susanne L Wilson, Jennifer A Harper, Jean S Payne, Diane Green, Paul Monaghan, Dragana Stanley, John A Donald, Kevin R Nicholas, Robert J Moore

**Affiliations:** 1 Australian Animal Health Laboratory, CSIRO Animal, Food and Health Sciences, 5 Portarlington Road, Geelong, Victoria, Australia; 2 School of Life and Environmental Sciences, Deakin University, Pigdons Road, Geelong, Victoria 3216, Australia; 3 Centre for Chemistry and Biotechnology, Deakin University, Geelong, Victoria 3216, Australia; 4 School of Medical and Applied Sciences, Central Queensland University, Rockhampton, QLD 4702, Australia

## Correction notice

The authors of ‘Transcriptome analysis of pigeon milk production – role of cornification and triglyceride synthesis genes’ [1] have advised the journal that Dragana Stanley was inadvertently omitted from the list of authors. The Competing interests, Authors’ contributions and Acknowledgements sections have been modified accordingly.

## Competing interests

The authors declare that they have no competing interests.

## Authors’ contributions

Conceived the project: MG, TC, VH and RM. Contributed to the formulation of ideas: JD, KN and PM. Carried out experimental work: MG, VH, JP, JH, DG and PM. Responsible for animal husbandry: SW. Analysed data: MG, DS and VH. Wrote the manuscript: MG. All authors read and approved the final manuscript.
