# Supplementary material for: Transcriptome analysis of pigeon milk production – role of cornification and triglyceride synthesis genes
Source: BMC Genomics. 2013 Mar 13;14:169. doi: 10.1186/1471-2164-14-169 (PMC3610128; doi:10.1186/1471-2164-14-169)
Supplement: Additional file 1: Table S1 — All differentially expressed probes in lactating crop compared to control crop. Table S2. Number of probes differentially expressed in female lactating crop compared to male lactating crop at the same lactation timepoint. [file 1471-2164-14-169-S1.docx]

# Additional tables

## Additional table 1 - The number of differentially expressed genes varies across lactation timepoints

| Time point | Number of down-regulated probes | Number of up-regulated probes | Total (% of all probes) |
| --- | --- | --- | --- |
| -8 (n = 10) | 0 | 0 | 0 (0.00) |
| -2 (n = 10) | 0 | 0 | 0 (0.00) |
| 0 (n = 14) | 4535 | 3937 | 8472 (17.21) |
| +2 (n = 10) | 11531 | 12491 | 24022 (48.80) |
| +10 (n = 4) | 473 (0.96) | 852 | 1325 (2.69) |

Differentially expressed probes in lactating crop compared to control crop (Welch t-test with Benjamini and Hochberg post-hoc test, *p* = 0.05). Control n = 13.

## Additional table 2 - Differences in sex-specific crop gene expression

| Timepoint | Number of differentially expressed probes (% of probes on microarray) |
| --- | --- |
| Ctrl | 0 (0.00) |
| -8 | 66 (0.13) |
| -2 | 34 (0.07) |
| 0 | 305 (0.62) |
| +2 | 0 (0.00) |
| +10 | 0 (0.00) |

Differentially expressed probes in female crop tissue as compared to male crop tissue at the same lactation timepoint.
